# Manejo Exitoso de la Pérdida de Efecto Fin de Dosis con Eptinezumab: Enseñanzas de un Caso de Migraña Crónica Refractaria a Dos Anticuerpos Anti-CGRP Subcutáneos

**DOI:** 10.31083/RN38974

**Published:** 2025-03-12

**Authors:** Marcos Polanco Fernández, Lara Gangas Barranquero, Vicente González-Quintanilla, Jorge Madera Fernández, Julio Pascual

**Affiliations:** ^1^Servicio de Neurología, Hospital Universitario Marqués de Valdecilla, Universidad de Cantabria e IDIVAL, 39008 Santander, España

**Keywords:** Deterioro fin de dosis, CGRP, anticuerpo monoclonal anti-CGRP, migraña, eptinezumab, eptinezumab, CGRP, monoclonal CGRP antibody, migraine, wearing-off

## Abstract

**Introducción:**

Los anticuerpos monoclonales contra el péptido relacionado con el gen de la calcitonina (CGRP) han supuesto una revolución en el tratamiento de la migraña, aunque un tercio de los pacientes no responden a estos fármacos. Una de las causas emergentes de no respuesta aparente podría ser la pérdida del efecto fin de dosis, como demuestra el caso de nuestra paciente.

**Caso Clínico:**

Mujer de 36 años con diagnóstico de migraña con aura desde la infancia y evolución a migraña crónica con cefalea diaria en los últimos 5 años asociando abuso de medicación y múltiples fallos a preventivos orales, toxina botulínica y dos anticuerpos contra el CGRP (erenumab y galcanezumab). Tras el inicio de eptinezumab trimestral, presenta una importante mejoría en el número de días de cefalea al mes durante las primeras 8 semanas, pero experimenta un claro deterioro fin de dosis en el tercer mes durante los dos primeros ciclos de tratamiento. Por ello, se ajusta su administración a cada 8 semanas con un excelente control de la frecuencia de su migraña.

**Conclusión:**

Los anticuerpos anti-CGRP pueden presentar pérdida de efecto fin de dosis, siendo necesaria su identificación para realizar ajuste posológico individualizado y evitar así, de manera errónea, etiquetarlo como un fracaso terapéutico. Nuestro caso demuestra, además, que los pacientes con migraña crónica refractaria a dos anticuerpos pueden responder a un tercer fármaco, en este caso a eptinezumab por vía intravenosa.

## Abstract (en)

**Introduction:**

Monoclonal antibodies against calcitonin gene-related peptide (CGRP) have revolutionized migraine treatment, though one-third of patients do not respond to these medications. One of the emergent reasons for this apparent lack of response could be a wearing-off effect, as the case of our patient illustrates.

**Clinical Case:**

A woman aged 36 years with the diagnosis of migraine with aura since childhood, which transforms to chronic migraine and daily headache, with analgesic overuse for the past 5 years. She failed to respond to multiple oral preventatives, botulinum toxin, and two CGRP antibodies (erenumab and galcanezumab). After initiating quarterly eptinezumab, she noticed a relevant improvement in the number of headache days per month for the first 8 weeks but experienced a clear wearing-off effect during the third month of treatment, for two consecutive treatments. We altered administration to every 8 weeks, which better controlled her migraine frequency.

**Conclusions:**

CGRP antibodies can show a wearing-off effect, which should be identified in order to plan individualized treatment and avoid an incorrect interpretation as therapeutic failure. Our case also shows that patients with chronic migraine refractory to two antibodies can respond to a third CGRP antibody, in this case, intravenous eptinezumab.

## 1. Introducción

La migraña crónica es una enfermedad que produce un gran impacto a nivel socioeconómico, en la calidad de vida de los pacientes y plantea un gran reto terapéutico. La aparición de los anticuerpos monoclonales contra el péptido relacionado con el gen de la calcitonina (CGRP) ha supuesto una revolución en el manejo de los pacientes sin respuesta a los fármacos orales clásicos [1]. Existen actualmente cuatro anticuerpos anti-CGRP. Tres son de administración subcutánea: erenumab mensual, fremanezumab mensual o trimestral, y galcanezumab mensual. Eptinezumab, el único intravenoso, es de administración trimestral. Casi dos tercios de los pacientes con migraña crónica o de alta frecuencia que no habían respondido a los fármacos preventivos orales y a la toxina botulínica tipo A responden a estos anticuerpos [1, 2]. El manejo de los pacientes refractarios a alguno de estos anticuerpos no es sencillo. Alrededor de un tercio de estos pacientes refractarios parecen responder si se les cambia de anticuerpo anti-CGRP, si bien no disponemos de estudios reglados. En la práctica clínica, además, algunos pacientes refieren una pérdida del efecto de fin de dosis previo a la siguiente administración del fármaco, algo que también vemos en el tratamiento preventivo de la migraña con toxina botulínica y que los pacientes interpretan como un fallo de tratamiento [3, 4, 5, 6, 7, 8]. Presentamos el caso de una mujer con diagnóstico de migraña crónica refractaria con fallo a múltiples tratamientos preventivos orales, toxina botulínica y dos anticuerpos anti-CGRP subcutáneos que respondió a la infusión intravenosa con eptinezumab, pero con deterioro fin de dosis que fue manejado exitosamente adelantando su administración. La adhesión a las directrices CARE garantiza que este informe de caso cumpla con los más altos estándares de claridad y exhaustividad. La lista de verificación se proporciona en los materiales suplementarios como referencia (**Material Suplementario-CARE-checklist-Spanish-2013**).

## 2. Caso Clínico

Se trata de una mujer de 36 años, con único antecedente reseñable de síndrome ansioso-depresivo, con comienzo de cefaleas episódicas típicamente migrañosas con aura visual a los 7 años de edad. La paciente es referida desde otro hospital donde había consultado hacía 5 años por empeoramiento de la frecuencia de los episodios de migraña. El dolor se había hecho diario/prácticamente diario, era habitualmente invalidante, pulsátil y se acompañaba de intenso y fotofobia. Su exploración neurológica y una resonancia magnética (RM) de cráneo eran normales. Tomaba anti-inflamatorios no esteroideos casi a diario y triptanes más de 15 días al mes. Como tratamiento preventivo había fracasado con dosis terapéuticas de metoprolol, topiramato, zonisamida, amitriptilina, lamotrigina y toxina botulínica tipo A. En su hospital había sido tratada con erenumab (inicialmente 70 y después 140 mg) y galcanezumab. Con ambos anticuerpos había tenido una aparente respuesta inicial transitoria, que se perdió a los pocos meses en ambos casos por lo que fueron retirados.

**Fig. 1.**
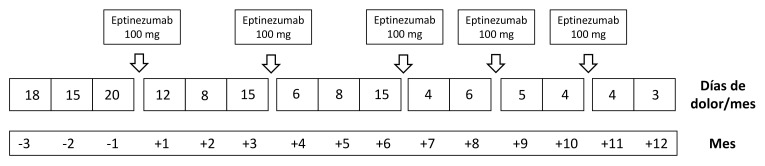
**Evolución de la frecuencia mensual de las crisis de migraña tres meses antes y un año después de la administración de eptinezumab 100 mg**. Nótese que la reducción en la frecuencia de las crisis de migraña tras eptinezumab es patente en los dos meses siguientes a su administración.

En la primera evaluación en nuestra consulta, la paciente presentaba más de 20 días de dolor al mes, la mayoría compatibles con migrañas con dolor intenso. Iniciamos tratamiento con ácido valproico sin clara respuesta (persistían crisis de migraña entre 15 y 20 días al mes), por lo que solicitamos un uso especial de eptinezumab a la dosis de 100 mg trimestral por vía intravenosa. Con este fármaco, la respuesta inicial era excelente en los dos primeros meses de tratamiento, en los que se objetivó una clara reducción en el número, en la intensidad de las crisis y en el consumo de medicación sintomática. Sin embargo, este beneficio se perdía (durante dos trimestres consecutivos) en el tercer mes tras la infusión, algo que la paciente interpretaba como un fracaso del tratamiento. Solicitamos autorización para administrar eptinezumab al final del segundo mes y, con esta posología de administración, la paciente permanece actualmente con una frecuencia de 3–5 días de cefalea al mes, menos intensos y con buena respuesta a analgesia convencional, lo que le permite realizar una vida personal y laboral normal (Fig. 1).

## 3. Discusión

De nuestra experiencia con esta paciente se pueden extraer dos enseñanzas principales. En primer lugar, que pacientes con migraña crónica refractarios a dos anticuerpos pueden responder a un tercero. En estos casos hemos de tener en cuenta que son pacientes desesperados y con una nefasta calidad de vida y que han agotado todos los tratamientos, por lo que parece justificado intentar todas las opciones disponibles, en este caso anticuerpos anti-CGRP. Dentro de estos anticuerpos existen algunos datos que sugieren, como ocurrió en esta paciente, que eptinezumab, probablemente por su administración intravenosa, pudiera ser útil en pacientes refractarios a otros anticuerpos [5]. En segundo lugar, nuestra paciente es un claro ejemplo del fenómeno de pérdida de respuesta fin de dosis [3, 4, 5, 6, 7, 8], que puede malinterpretarse, y tal vez podría incluso haber explicado en parte el aparente fracaso con erenumab y galcanezumab, como una ausencia de respuesta si, como es habitual, revisamos al paciente al tercer mes de tratamiento. Los datos que disponemos sobre el fenómeno de deterioro fin de dosis son discrepantes y no concluyentes. Aunque algunos autores han afirmado que este fenómeno no ocurre con los anticuerpos anti-CGRP [9, 10], estudios más recientes, aunque con diseños muy heterogéneos, cifran la tasa de deterioro fin de dosis entre el 9 y casi el 40% (Tabla 1) [6, 7, 8]. En estos casos con sospecha de deterioro fin de dosis es mandatorio conocer la frecuencia y distribución exacta de las crisis mes a mes para poder identificar y tratar este fenómeno de pérdida de respuesta fin de dosis. Nuestra paciente demuestra, además, que la frecuencia de la administración (y también probablemente las dosis) deben de ser individualizados y flexibilizados en algunos pacientes como el que presentamos para tratar de optimizar la respuesta a estos fármacos y mejorar así su calidad de vida.

**Tabla 1.** **Trabajos que analizan el deterioro fin de dosis con pacientes con migraña en tratamiento con anticuerpos anti-CGRP**.

Autores	Fármaco	N	Wearing-off
George N. *et al*., 2020 [8]	Erenumab	190	17 (9%)
Ailani J *et al*., 2022 [6]	Galcanezumab	903	5,2% to 8,2%
Dapkutė A *et al*., 2022 [7]	Erenumab	145	37,8%

CGRP, péptido relacionado con el gen de la calcitonina.

## 4. Conclusiones

Los anticuerpos anti-CGRP pueden presentar pérdida de efecto fin de dosis. Es necesario su identificación para realizar ajuste posológico individualizado, por ejemplo, acortando el intervalo de administración, y evitar así, de manera errónea, interpretarlo como un fracaso terapéutico. El caso de nuestro paciente nos enseña, además, que los pacientes con migraña crónica refractaria a dos anticuerpos pueden responder a un tercer fármaco, en este caso a eptinezumab por vía intravenosa.

## Disponibilidad de Datos y Materiales

Los detalles de este trabajo están a disposición de los investigadores que lo soliciten al autor responsable.

## Contribuciones de los Autores

MPF, LGB, VGQ, JMF y JP recogida de datos. JP elaboración del manuscrito. Todos los autores contribuyeron a los cambios editoriales en el manuscrito. Todos los autores leyeron y aprobaron el manuscrito final. Todos los autores han participado lo suficiente en el trabajo y han acordado ser responsables de todos los aspectos del mismo.

## Aprobación Ética y Consentimiento Informado

El estudio fue aprobado por el Comité de Etica de Cantabria (2023/3054). La paciente objeto de esta publicación dio su consentimiento informado para su publicación y su manejo cumple con los requisitos de la declaración de Helsinki.

## Agradecimientos

Los autores agradecen a la paciente su colaboración en este estudio.

## Financiación

Esta investigación no recibió financiación externa.

## Material Suplementario

El material suplementario asociado con este artículo se puede encontrar en la versión en línea, en https://doi.org/10.31083/RN38974.
