# Supplementary material for: Successful Management of Wearing-off effect with Eptinezumab: Lessons from a case with Chronic Migraine Refractory to Two Subcutaneous CGRP Antibodies
Source: Rev Neurol. 2025 Mar 12;80(2):38974. [Article in Spanish] doi: 10.31083/RN38974 (PMC11973719; doi:10.31083/RN38974)
Supplement: Supplementary file 1 [file 1576-6578-80-2-38974-s1.pdf]

CARE case report guidelines

Lista de comprobación CARE (2013) de la información a incluir al Redactar un informe de caso

(cc) BY-NC-ND

Asunto	Elemento	Descripción del elemento de la lista de comprobación	Informado en la página
**Título**	1	Las palabras “informe de caso” deben aparecer en el título junto con lo más interesante de este caso.	1
**Palabras clave**	2	Los elementos clave de este caso en 2 - 5 palabras clave.	1
**Resumen**	3a	Introducción— ¿Qué es único en este caso? ¿Qué aporta de nuevo a la literatura médica?	1
	3b	Los principales síntomas del paciente y los hallazgos clínicos importantes.	1
	3c	Los principales diagnósticos, intervenciones terapéuticas y resultados	1
	3d	Conclusión— ¿Cuáles son las principales lecciones que se pueden extraer de este caso?	1
**Introducción**	4	Breve resumen de los antecedentes de este caso haciendo referencia a la literatura médica pertinente.	2
**Información del paciente**	5a	Información demográfica (como edad, sexo, origen étnico, profesión).	2
	5b	Principales síntomas de paciente (sus principales molestias).	2
	5c	Historial médico, familiar y psicosocial que incluya la dieta, el estilo de vida y la información genética pertinente.	2
	5d	Enfermedades concomitantes pertinentes, incluyendo intervenciones anteriores y sus resultados	2
**Hallazgos clínicos**	6	Describir los hallazgos pertinentes de la exploración física (EF).	2
**Calendario**	7	Describa hitos importantes relacionados con sus diagnósticos e intervenciones (tabla o figura)	2
**Evaluación diagnóstica**	8a	Métodos diagnósticos (como la EF, analíticas, técnicas de obtención de imágenes, cuestionarios).	2
	8b	Problemas para el diagnóstico (como económicos, lingüísticos o culturales).	NA
	8c	Razonamiento diagnóstico, incluidos otros posibles diagnósticos tenidos en cuenta	2
	8d	Características de pronóstico (como los estadios en oncología) cuando proceda.	NA
**Intervención terapéutica**	9a	Tipos de intervención (como farmacológica, quirúrgica, preventiva, autocuidados).	2
	9b	Administración de la intervención (como dosis, concentración, duración).	2
	9c	Cambios en la intervención (con justificación).	2
**Seguimiento y resultados**	10a	Resultados evaluados por el médico y por el paciente.	2
	10b	Resultados importantes de la prueba de seguimiento	2
	10c	Observancia de la intervención y tolerabilidad a la misma (¿cómo se ha evaluado?).	2
	10d	Acontecimientos adversos e imprevistos.	2
**Discusión**	11a	Puntos fuertes y limitaciones en el manejo de este caso.	2,3
	11b	Discusión de la literatura médica pertinente.	3
	11c	Justificación de las conclusiones (incluida la evaluación de las posibles causas)	2,3
	11d	Las principales lecciones que se pueden extraer de este informe de caso.	3
**Perspectiva del paciente**	12	¿Comunicó el paciente su perspectiva o experiencia? (Incluir siempre que sea posible).	NA
**Consentimiento informado**	13	¿Dio su consentimiento informado el paciente? Facilítelo si se le solicita.	Sí [ ] X No [ ]
